# Tight Boots

**Published:** 1869-02

**Authors:** 


### TIGHT BOOTS.

Many a man has started from a shoe store feeling that he has had a "nice fit," who by the time he has walked two hundred yards has wished vehemently that both boot and maker were at the very bottom of the nethermost sea. Recently a death is reported within a few hours after marriage as the result of too tight-fitting boots of the bridegroom, he having been thrown into convulsions. A previous suggestion is repeated as a means of making new boots feel as comfortable as an "old shoe," and it never fails. Put on two pairs of thick winter or summer stockings, and any boot which can be drawn on with any reasonable effort will be comfortably loose when put on over but one pair. The reader need but try this once to feel satisfied of the practical value of the suggestion.

